# A new species of pterodectine feather mites (Acarina, Analgoidea, Proctophyllodidae) from the Little Spiderhunter *Arachnothera longirostra* (Passeriformes, Nectariniidae) in Meghalaya, India

**DOI:** 10.3897/zookeys.425.7587

**Published:** 2014-07-10

**Authors:** Ioana Cristina Constantinescu, Gabriel Chişamera, D. Khlur B. Mukhim, Costică Adam

**Affiliations:** 1 “Grigore Antipa” National Museum of Natural History, Sos. Kiseleff no.1, 011341 Bucharest 1, Romania; 2 Zoology Department, Lady Keane College, 793001 Shillong, Meghalaya, India

**Keywords:** Pterodectinae, *Pedanodectes angustilobus*, new species, systematics

## Abstract

The article describes a new species of the feather mite subfamily Pterodectinae from the Little Spiderhunter *Arachnothera longirostra* Temminck, 1826 (Passeriformes, Nectariniidae) in India. *Pedanodectes angustilobus* **sp. n.** differs from all known *Pedanodectes* species by having opisthosomal lobes short, at base wider than long, roughly rounded apically in males, and strongly elongated and narrowed lobar region with wide terminal appendages in females. A key to species of the genus *Pedanodectes* is presented.

## Introduction

Feather mites are commensals or ectoparasites permanently living on birds. In India, the diversity of feather mites is poorly investigated, and only 26 species have been mentioned so far in various taxonomic papers (Oudemans 1904, Bonnet 1924, Gaud and Mouchet 1963, Atyeo et al. 1972, Gaud 1972, McClure and Ratanaworabhan 1973, Gaud and Atyeo 1976, 1987, Santana 1976, Peterson et al. 1980, D'Souza and Jagannath 1982, Atyeo 1984, Gaud et al. 1985, 1988, Dabert and Ehrnsberger 1998, Dabert 2003, Mironov et al. 2002, Putatunda et al. 2004, Constantinescu et al. 2014). Atyeo (in McClure and Ratanaworabhan 1973) reported five new species of feather mites collected in Asia from the host *Arachnothera longirostra* (including *Pedanodectes* species), but unfortunately this material has never been described.

In this paper, we describe a new species of *Pedanodectes* found on *Arachnothera longirostra* in India and we present a key for all species of this genus.

## Materials and methods

The material used in the present paper was collected in Meghalaya (India), in January 2014. The birds were trapped by means of ornithological mist nets, identified and visually checked for the presence of mites and, after mites were collected, released back to the wild. Mite specimens were placed in tubes with 95% ethanol. Later, in the laboratory, the mite specimens were cleared in lactic acid and mounted on microscope slides in Hoyer’s medium. Drawings were made using an Olympus CX21 microscope, using a camera lucida drawing device.

The bird specimens were identified according to Rasmussen and Anderton (2012) and Grimmett et al. (2011), and the taxonomy of the birds used in the present paper follows Clements et al. (2013). The description of new species is given according to the current format used for species of pterodectine mites (Mironov and Fain 2003, Valim and Hernandes 2006, Mironov 2006, Mironov et al. 2008). The body chaetotaxy of mites follows that of Griffiths et al. (1990) with the modifications by Norton (1998) concerning coxal setae, and the chaetotaxy of legs follows Gaud and Atyeo (1996). The measuring techniques of particular structures used in the present paper were recently described by Mironov and Proctor (2009). We give the full set of measurements for the holotype (male) and a range of measurements for all corresponding paratypes. All measurements are in micrometers (μm). The holotype and all paratypes of the new species are deposited in the Acarological Collection of the “Grigore Antipa” National Museum of Natural History, Bucharest, Romania.

## Results

### Family Proctophyllodidae Trouessart & Mégnin, 1884
Subfamily Pterodectinae Park & Atyeo, 1971
Genus *Pedanodectes* Park & Atyeo, 1971

The genus currently includes six species, associated with birds of the order Passeriformes (families Nectariniidae, Malaconotidae, Cisticolidae and Platysteiridae) in Africa (see Table 1).

**Table 1.** *Pedanodectes* species and their host associations.

Mite species	Host species	Host family	Location	References
*Pedanodectes hologaster* (Gaud, 1953)	*Chalcomitra senegalensis* (Linnaeus)*, *Chalcomitra fuliginosa* (Bechstein)	Nectariniidae	Central African Republic	Gaud 1953; Park and Atyeo 1971
*Pedanodectes andrei* (Till, 1954)	*Tchagra senegalus* (Linnaeus)	Malaconotidae	Mozambique	Till 1954
*Pedanodectes mesocaulus* (Gaud & Mouchet, 1957)	*Deleornis fraseri cameroonensis* (Bannerman)	Nectariniidae	Cameroon	Gaud and Mouchet 1957
*Pedanodectes marginatus* Mironov & Kopij, 1997	*Camaroptera brachyura* (Vieillot)	Cisticolidae	South Africa	Mironov and Kopij 1997
*Pedanodectes latior* Mironov & Kopij, 1997	*Platysteira peltata* Sundevall	Platysteiridae	South Africa	Mironov and Kopij 1997
*Pedanodectes blaszaki* Mironov, 2008	*Cinnyris cupreus* (Shaw)	Nectariniidae	South Africa	Mironov 2008
*Pedanodectes angustilobus* sp. n.	*Arachnothera longirostra* (Latham)	Nectariniidae	India	Present paper

* - Type host

#### Pedanodectes angustilobus

Taxon classificationAnimaliaAstigmataProctophyllodidae

Constantinescu sp. n.

http://zoobank.org/186A8195-8BE0-4212-A864-0FB3A224DC66

**Figure 1.**
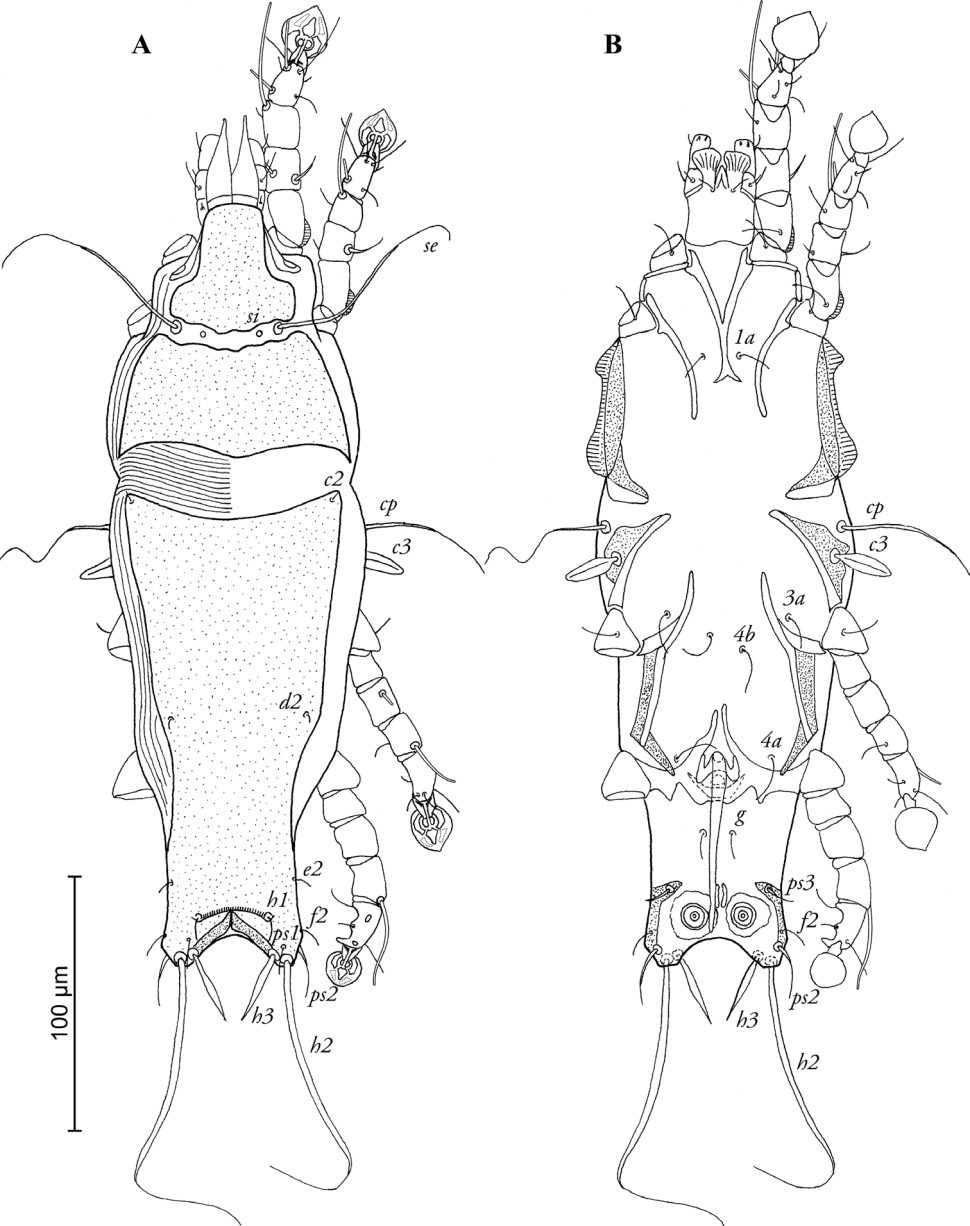
*Pedanodectes angustilobus* sp. n., male holotype: **A** dorsal view of idiosoma **B** ventral view of idiosoma.

Figs 1 [Fig F2] [Fig F3] –4

##### Type material.

Male holotype (ANA256), 3 male (ANA257, ANA258, ANA259) and 4 female (ANA260, ANA261, ANA262, ANA263) paratypes from the Little Spiderhunter *Arachnothera longirostra* Temminck, 1826 (Passeriformes, Nectariniidae); **INDIA:** Meghalaya, Jaintia Hills, Shnongrim village, (25°21'12.36"N, 92°31'3.06"E); 1151 m alt; 24.01.2014, collector Costică Adam.

##### Description.

MALE (Figs 1A, B; 3A–D; holotype, range for 3 paratypes in parentheses): Length of idiosoma 316 (308–317), width 100 (100–104), length of hysterosoma 212 (207–212). Prodorsal shield divided into two parts by transversal band of soft tegument bearing setae *se* and *si*, antero-lateral extensions short and rounded, posterior margin slightly convex in median part, total length of shield 102 (102–106), greatest width 90 (84–90), surface without ornamentation (Fig. 1A). Scapular setae *se* separated by 38 (36–40). Humeral shields absent, setae *cp* situated ventrally, setae *c2* situated dorsally, in anterior angles of hysteronotal shield. Subhumeral setae *c3* lanceolate, 20 (19–20) × 6 (5–6). Length of hysteronotal shield from anterior margin to bases of setae *h2* 195 (199–203), greatest width in anterior part 88 (80–86), anterior margin concave, anterior angles acute, surface without ornamentation with strongly sclerotized transverse fold between *h1* setae. Opisthosomal lobes short, at base wider than long, roughly rounded apically, lobar apices bearing setae *h2*. Terminal cleft almost semicircular, with narrow membranous margin in anterior part, length of terminal cleft 10 (12–13). Supranal concavity absent. Hysteronotal setae *c1, d1, e1* absent; setae *h3* narrowly lanceolate, length 24 (24–25), greatest width 6 (4–6); setae *h2* represented by macrosetae, length 130 (132–140), greatest width 5 (4–5); setae *ps2* slightly thickened, 18 (12–16) long; setae *ps1* filiform, minute, length 6 (5–6), situated slightly anterior to bases of setae *h3* and *h2*, approximately equidistant from inner and outer margins of opisthosomal lobe. Dorsal measurements: *se*-*c2* 72 (66–70), *c2*-*d2* 92 (90–104), *d2*-*e2* 68 (60–66), *e2*-*h3* 32 (32–34), *h1-h3* 10 (10–12), *h2-h2* 38 (38–42), *h3*-*h3* 26 (26–28), *ps2*-*ps2* 50 (46–49). Epimerites I fused into a Y, sternum about ½ of the total length of epimerites, posterior end of sternum with pair of postero-lateral extensions not connected to epimerites II. Epimerites II short, not extending to level of sejugal furrow. Coxal fields I–III open, without wide sclerotized areas. Epimerites IVa long, their anterior ends extending to midlevel of epimerites IV, their basal parts connected by semicircular transverse sclerite (supposedly genital shield) and almost completely encircling genital apparatus (Fig. 1B). Genital arch 11 (11–12) long, 14 (13–14) wide, basal sclerite of genital apparatus large, poorly sclerotized; aedeagus long, extending almost to anterior margin of terminal cleft, length of aedeagus from its anterior bend to tip 74 (72–78). Genital papillae indistinct, adanal shields absent. Anal suckers 11 (11–12) in diameter, corolla without indentations. Opisthoventral shields occupying lateral margin of opisthosoma, with narrow inner projection bearing seta *ps3*. Ventral measurements: *3a*-*4b* 22 (25–30), *4b-4a* 50 (44–50), *4a-g* 24 (24–28), *g-ps3* 32 (28–31), *ps3-ps3* 40 (32–38), *ps3-h3* 24 (23–24).

Legs I slightly longer and thicker than legs II, femora I and II with ventro-basal crests (Fig. 3A, B). Seta *mG*II strongly thickened in basal half. Tarsus IV 18 (19–22) long, with apical claw and with small apico-ventral extensions bearing seta *r* and *w*; setae *d, e* button-like, seta *d* bigger than *e*, situated in basal and apical parts of segment, respectively (Fig. 3D). Length of solenidia: *ω1*I 15 (14–15), *ω1*II 12 (13–16), *φ*I 42 (34–46), *φ*II 29 (32–34), *φ*III 22 (22–24), *φ*IV 32 (28–32).

**Figure 2. F2:**
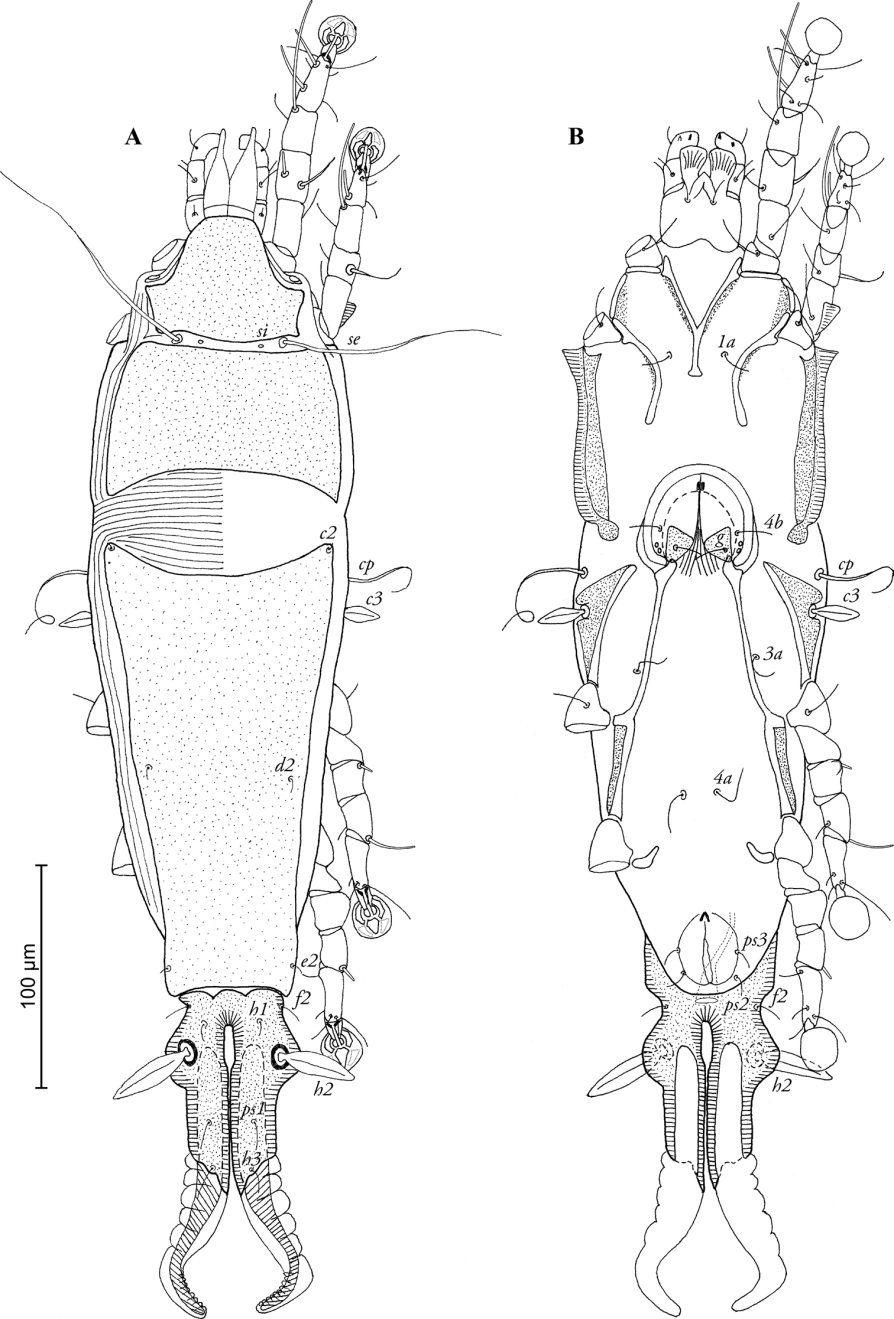
*Pedanodectes angustilobus* sp. n., female paratype: **A** dorsal view of idiosoma **B** ventral view of idiosoma.

FEMALE (Figs 2A, B; 4A–E; range for 4 paratypes): Length of idiosoma excluding terminal appendages 436–456, width 118–124, and length of hysterosoma 312–340. Prodorsal shield divided into two parts by transversal band of soft tegument, bearing setae *se* and *si*, antero-lateral extensions short and angular, posterior margin concave, total length of shield 118–122, greatest width 104–112, surface without ornamentation (Fig. 2A). Scapular setae *se* separated by 48–50. Humeral shields absent, setae *cp* situated ventrally, setae *c2* situated dorsally, in anterior angles of hysteronotal shield. Subhumeral setae *c3* lanceolate, 17–20 × 5–6. Hysteronotal shield divided into anterior hysteronotal shield and lobar shield. Anterior hysteronotal shield roughly rectangular, with anterior margin concave, greatest length 204–208, greatest width in anterior part 100–106, surface without ornamentation. Lobar shield elongated and narrowed, with well-developed lateral extensions bearing setae *h2*, length of lobar shield 86–92, width at level of setae *h2* 58–62. Terminal cleft narrow, almost parallel-sided, with narrow elliptical part in anterior third, length 72–76, greatest width 3–4. Supranal concavity absent. Terminal appendages wide, their width in basal half similar to that of lobes. Hysteronotal setae *c1, d1, e1* absent; setae *h1* situated on lobar shield at level of anterior end of terminal cleft, setae *h2* spindle-shaped, without terminal filaments, 38–40 × 6–9. Setae *ps1* situated approximately equidistant from outer and inner margins of opisthosomal lobes, setae *h3* 6–8 long. Dorsal measurements: *se*-*c2* 88–94, *c2*-*d2* 100–112, *d2*-*e2* 86–94, *e2*-*h2* 36–44, *h2-h3* 48–52, *h1-h2* 16–22, *h1*-*h1* 18–22, *h2*-*h2* 36–40, *h3*-*h3* 20–24. Epimerites I fused as a Y, posterior end of sternum with small rounded lateral extensions not reaching epimerites II. Coxal fields I, II open, without heavily sclerotized areas, outer margins of epimerites I and II with narrow sclerotized areas (Fig. 2B). Epimerites IVa rudimentary. Translobar apodemes of opisthosomal lobes present, fused to each other anterior to terminal cleft. Epigynum horseshoe-shaped, outer margin with lateral extensions, greatest width 52–60. Head of spermatheca as in Fig. 4E, primary spermaduct with three different enlargements: ball-like enlargement near very head of spermatheca; moderate enlargement with verrucous external surface in proximal 1/5 of this duct; conical enlargement at copulatory opening. Secondary spermaducts short, not longer that ball-like enlargement of primary spermaduct. Copulatory opening situated ventral, posterior to anal opening. Distance between pseudanal setae: *ps2-ps2* 24–286, *ps3-ps3* 24–29, *ps2-ps3* 10–14.

Legs I longer and thicker than legs II, femora II with ventro-basal crests, genua III with dorso-basal crest (Fig. 4A–C). Genual setae *mG*I and *mG*II noticeably thickened in basal half. Length of solenidia: *ω1*I 17–18, *ω1*II 13–16, *φ*I 45–56, *φ*II 32–38, *φ*III 22–27, *φ*IV 7–8.

**Figure 3. F3:**
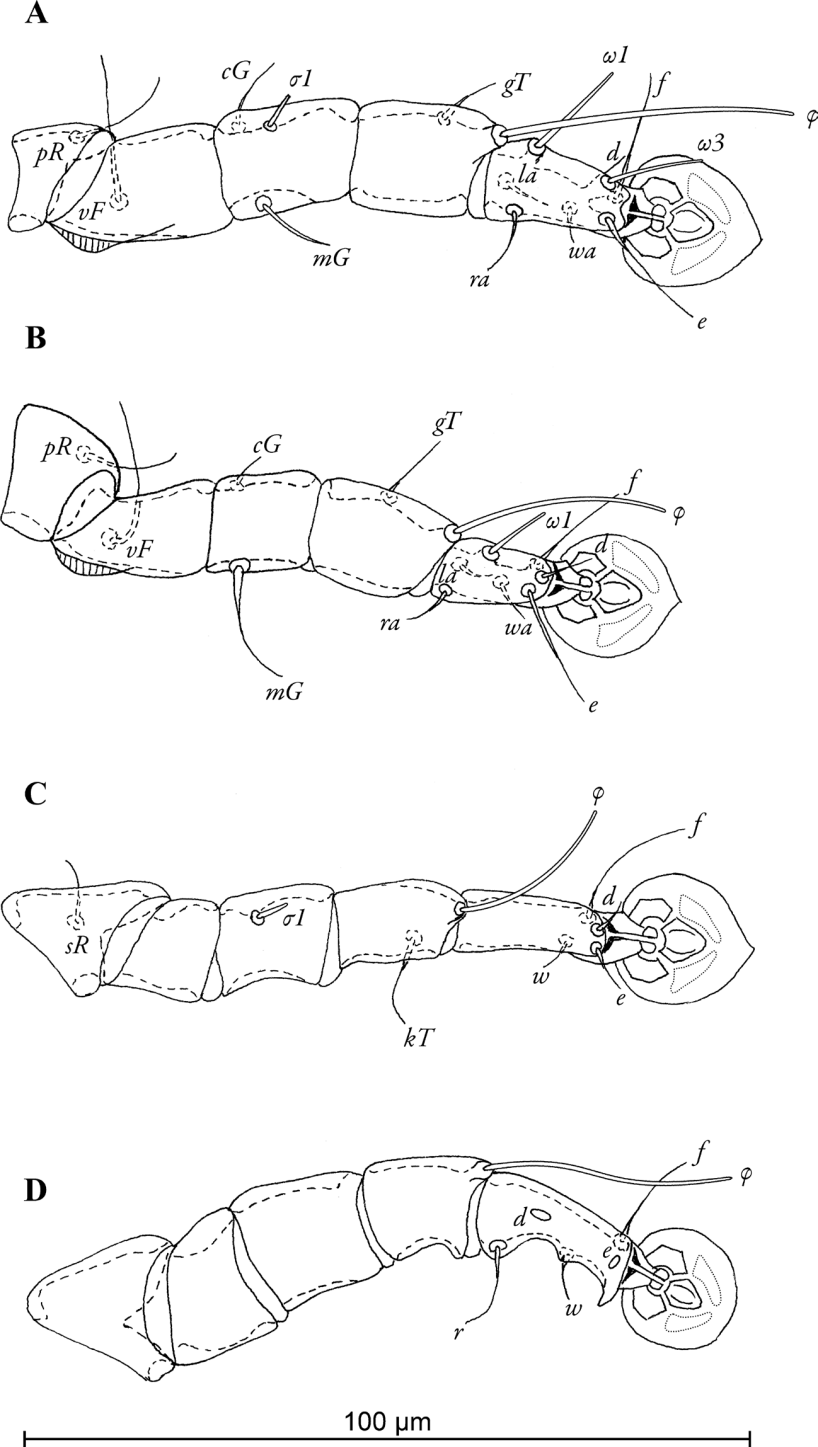
*Pedanodectes angustilobus* sp. n., details: **A–D** legs I–IV of male, respectively, dorsal view.

**Figure 4.**
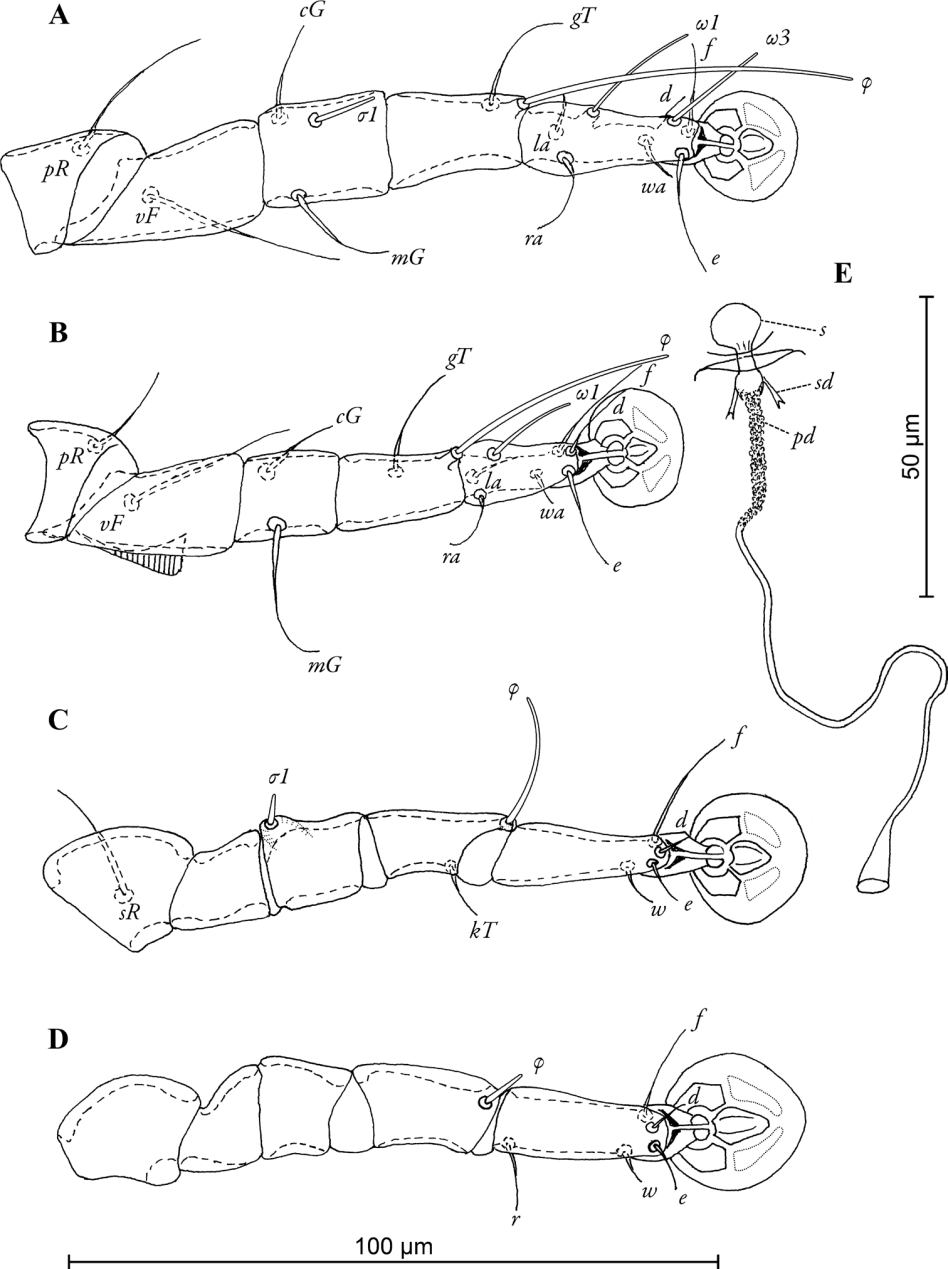
*Pedanodectes angustilobus* sp. n., details: **A–D** legs I–IV of female, respectively **E** spermatheca and spermaducts, dorsal view. Abbreviations: pd - primary spermaduct; s - spermatheca; sd - secondary spermaduct.

##### Etymology.

The specific epithet refers to the narrowed lobar region of the female, and it is an adjective in the nominative singular.

##### Remarks.

*Pedanodectes angustilobus* sp. n. clearly differs from all previously described *Pedanodectes* species because its males have well expressed opisthosomal lobes, and females have elongated and narrowed lobar region, with wide terminal appendages. Among previously known species of the genus, males of *Pedanodectes angustilobus* sp. n. appear to be closest to that of *Pedanodectes mesocaulus* (Gaud & Mouchet, 1957) from *Deleornis fraseri cameroonensis* (Bannerman, 1921) (Passeriformes: Nectariniidae). Males of both species have setae *ps1* situated antero-lateral to the adanal suckers, epimerites I fused into Y, similar shape of opisthoventral shields and epimerites IVa with narrow anterior projections. Males of *Pedanodectes angustilobus* sp. n. are easily to distinguish from those of *Pedanodectes mesocaulus* by the following features: epimerites IVa have free the anterior projections and are connected posterior to the genital arch by a transversal sclerite, the tip of aedeagus does not extend beyond the posterior margin of idiosoma. In males of *Pedanodectes mesocaulus*, the anterior projections of epimerites IVa are fused forming a pregenital sclerite while the transverse sclerite connecting the bases of these epimerites is absent, the tip of aedeagus extends beyond the posterior margin of idiosoma. Females of the new species are clearly different from those of the other species of the genus by the following unique combination of characters: the lobar region has the same width in anterior and posterior part; the terminal cleft is parallel-sided, with the margins almost touching, except for the anterior one third; and the terminal appendages are thick, their basal half is approximately as wide as opisthosomal lobes. In females of the other *Pedanodectes* species, the lobar region in the anterior part is wider than in the posterior part and the terminal appendages are narrower than lobes. The terminal cleft in females of the other species has the following shape: with the lateral margins parallel and almost touching in *Pedanodectes andrei* and *Pedanodectes mesocaulus*, with lateral margins sinuous and almost touching in certain parts in *Pedanodectes marginatus* and *Pedanodectes latior*; as a narrow inverted V in *Pedanodectes blaszaki*, and as a narrow inverted U in *Pedanodectes hologaster*.

## Discussion

According to the diagnosis of the genus *Pedanodectes*, some authors considered that males of this genus practically have no opisthosomal lobes and setae *ps3* are usually situated lateral or postero-lateral to the adanal suckers (Gaud and Atyeo 1996, Mironov 2008, Hernandes and Valim 2014). However in the original definition of this genus, Park and Atyeo (1971), apparently based on a some undescribed material they had on the hand, mentioned that the presence of weakly developed opisthosomal lobes and the position of setae *ps3* antero-lateral to the adanal suckers can be found in some species. The new species found on *Arachnothera longirostra* in India and described in this paper demonstrates the example of a *Pedanodectes* species having distinct opisthosomal lobes and setae *ps3* situated antero-lateral to the anal suckers in males.

### Key to males of *Pedanodectes*

**Table:** 

1	Epimerites I fused V-likely	2
–	Epimerites I fused Y-likely	4
2	Postero-lateral extensions of epimerites I connected to epimerites II	*Pedanodectes latior*
–	Postero-lateral extensions of epimerites I not connected to epimerites II	3
3	Tip of aedeagus extending beyond posterior margin of idiosoma	*Pedanodectes andrei*
–	Tip of aedeagus not extending beyond posterior margin of idiosoma	*Pedanodectes hologaster*
4	Postero-lateral extensions of epimerites I present	5
–	Postero-lateral extensions of epimerites I absent	*Pedanodectes blaszaki*
5	Postero-lateral extensions of epimerites I connected to epimerites II	*Pedanodectes marginatus*
–	Postero-lateral extensions of epimerites I not connected to epimerites II	6
6	Opisthosomal lobes present; tip of aedeagus not extending beyond posterior margin of idiosoma	*Pedanodectes angustilobus* sp. n.
–	Opisthosomal lobes absent; tip of aedeagus extending beyond posterior margin of idiosoma	*Pedanodectes mesocaulus*

### Key to females of *Pedanodectes*

**Table:** 

1	Width of terminal appendages in anterior half similar to that of opisthosomal lobes	*Pedanodectes angustilobus* sp. n.
–	Terminal appendages narrower than opisthosomal lobes	2
2	Lateral margins of terminal cleft spaced in some parts or on their entire length	3
–	Lateral margins of terminal cleft almost touching on their entire length	6
3	Prodorsal shield split into anterior and posterior pieces at level of scapular setae	*Pedanodectes blaszaki*
–	Prodorsal shield entire	4
4	Lateral margins of prodorsal shield entire	5
–	Lateral margins of prodorsal shield with deep incisions around setae *se*	*Pedanodectes latior*
5	Epimerites I fused V-likely	*Pedanodectes hologaster*
–	Epimerites I fused Y-likely	*Pedanodectes marginatus*
6	Posterior margin of anterior hysteronotal shield straight	*Pedanodectes andrei*
–	Posterior margin of anterior hysteronotal shield concave	*Pedanodectes mesocaulus*

## Supplementary Material

XML Treatment for Pedanodectes angustilobus
